# Predict Alzheimer’s disease using hippocampus MRI data: a lightweight 3D deep convolutional network model with visual and global shape representations

**DOI:** 10.1186/s13195-021-00837-0

**Published:** 2021-05-24

**Authors:** Sreevani Katabathula, Qinyong Wang, Rong Xu

**Affiliations:** Center for Artificial Intelligence in Drug Discovery, School of Medicine, Case Western Reserve University, 2103 Cornell Rd, Cleveland, OH 44106 USA

**Keywords:** Alzheimer’s disease, Hippocampus, Magnetic resonance imaging, 3D Convolutional neural network, Classification

## Abstract

**Background:**

Alzheimer’s disease (AD) is a progressive and irreversible brain disorder. Hippocampus is one of the involved regions and its atrophy is a widely used biomarker for AD diagnosis. We have recently developed DenseCNN, a lightweight 3D deep convolutional network model, for AD classification based on hippocampus magnetic resonance imaging (MRI) segments. In addition to the visual features of the hippocampus segments, the global shape representations of the hippocampus are also important for AD diagnosis. In this study, we propose DenseCNN2, a deep convolutional network model for AD classification by incorporating global shape representations along with hippocampus segmentations.

**Methods:**

The data was obtained from the Alzheimer’s Disease Neuroimaging Initiative (ADNI) and was T1-weighted structural MRI from initial screening or baseline, including ADNI 1,2/GO and 3. DenseCNN2 was trained and evaluated with 326 AD subjects and 607 CN hippocampus MRI using 5-fold cross-validation strategy. DenseCNN2 was compared with other state-of-the-art machine learning approaches for the task of AD classification.

**Results:**

We showed that DenseCNN2 with combined visual and global shape features performed better than deep learning models with visual or global shape features alone. DenseCNN2 achieved an average accuracy of 0.925, sensitivity of 0.882, specificity of 0.949, and area under curve (AUC) of 0.978, which are better than or comparable to the state-of-the-art methods in AD classification. Data visualization analysis through 2D embedding of UMAP confirmed that global shape features improved class discrimination between AD and normal.

**Conclusion:**

DenseCNN2, a lightweight 3D deep convolutional network model based on combined hippocampus segmentations and global shape features, achieved high performance and has potential as an efficient diagnostic tool for AD classification.

## Introduction

Alzheimer’s disease (AD) is a chronic neurological brain disorder characterized by memory loss and cognitive impairment [1]. Currently, there are no effective drug treatments available to cure AD, and the existing medicines can only ease symptoms or slow down its progression [2]. An early diagnosis of AD can help in determining its progression and also improve the quality of life of AD patients [3].

The current diagnosis of AD is made by clinical, neuropsychological, and neuroimaging assessments [4–9]. More recently, a variety of imaging modalities, including structural and functional magnetic resonance imaging (MRI) and positron emission tomography (PET) studies of cerebral metabolism, have shown characteristic changes in the brains of patients with AD [10–12]. MRI is considered the preferred neuroimaging examination for AD as it allows for high tissue contrast and accurate measurement of the 3-dimensional (3D) volume of brain structures, especially the size of the hippocampus and related regions [13]. Also, MRI has an exceptional spatial resolution, high accessibility, and good contrast. In recent years, extensive efforts have been done to identify biomarkers for structural changes and disease states of the brain with structural MRIs [12, 14].

Hippocampal atrophy measures from MRI are powerful biomarkers for monitoring AD progression [15, 16]. Features from the hippocampus have been studied for AD diagnosis-based structural MRIs [16–19]. Hippocampal visual features have been used in Support Vector Machines and Bayesian classifiers for AD diagnosis [17]. Hippocampal volume changes based on MRI were used as biomarkers and features for AD diagnosis [18]. However, methods based on volumetric analysis alone provide limited information about morphological changes that characterize the appearance and progression of AD. Recently, shape description/modeling methods have been proposed to analyze the development of AD [19]. Shape descriptors can be broadly divided into three categories: (1) histogram-based, (2) graph-based, and (3) transform-based shape descriptors. Some of the histogram-based descriptors are the shape spectrum, generalized shape distributions, probability density-based descriptors, 3D shape contexts, etc. [20]. The challenges here are to select discriminating shape functions and to robustly compute the dissimilarity between probability distributions [21]. The graph-based descriptors like medial axis, Reeb graph, skeletal graph, etc., capture geometrical and topological shape properties, but are more complex and difficult to be constructed and derived [22]. Transform-based descriptors mainly include Fourier transform descriptors, spherical space transform descriptor, spherical harmonics, etc. [23], and have been widely applied in the neuroimaging fields [24–26]. Gutman et al. performed shape analysis for the hippocampus using the spherical harmonic shape description [24]. Spherical harmonics requires spherical parameterization, a smooth mapping from the surface to a unit sphere [27]. But the shape descriptors based on Laplace Beltrami (LB) spectrum can be modeled for any Riemannian manifold, is isometry-invariant, and avoids pre-processing steps like mapping, registration, and alignment [28]. In [25], shape changes of MRI brain regions were studied using LB eigen value-based features.

Recently, deep learning (DL) techniques have been developed/used to find links between different parts of images and to identify disease-related patterns [29], including AD classification based on detailed hippocampus analysis using structural MRIs [30–33]. Deep learning models can extract the features directly from medical images to discover hidden representations. These models often outperform other machine learning approaches with the best results on image classification tasks. However, recent researches suggest that these models have limitations in recognizing objects by their global shapes or shape surfaces [34, 35]. And, it is well-known that the hippocampal shape changes are important biomarker in AD [15]. In this study, we hypothesize that integration of global shape representations with visual features of the hippocampus in a deep learning framework will improve the performance of AD classification.

We have recently developed a densely connected 3D convolution neural network (CNN) model (DenseCNN) for classifying AD from normal based on hippocampus segmentations [36]. DenseCNN is a lightweight model with fewer convolutional kernels, relatively simple structure, and fewer total parameters than other state-of-the-art deep learning models for AD classification. Recent studies demonstrated that deep convolution neural networks (DCNN) often did not capture global object shape features [34, 35]. DCNNs are able to encode the local shape features including local edge segments and relations. But, it is sensitive to how these local features fit together as a whole to represent global shape features. DCNNs trained for object recognition do not appear to represent global shape at all [34]. Thus, in this study, we propose DenseCNN2, a lightweight DenseCNN model with combined global shape and visual hippocampus segmentation features of hippocampus, to improve AD classification. Different from DenseCNN, DenseCNN2 is built not only on hippocampus segmentations but also their global shape representations. We demonstrated that DenseCNN2 performed better than DenseCNN and other state-of-the-art methods.

## Materials and method

### Data

The MRI Data was obtained from the ADNI (http://adni.loni.usc.edu) and the data is T1-weighted structural from initial screening or baseline, including ADNI 1,2/GO and 3. Hippocampus segmentation was performed on this MRI data. After the segmentation, data contained 326 AD subjects and 607 control normal (CN) subjects, totaling 933 hippocampus segmentations. This work is aimed at classifying AD vs. CN. The demographic information of the subjects is provided in Table 1.

**Table 1:** Demographic characteristics of the subjects from ADNI database (age and years of education are given as mean (standard deviation))

	AD (***N***=326)	CN (***N***=607)	***P*** value
**Gender (% female)**	47.54	54.36	0.054
**Age**	74.9 (7.6)	74.4 (7.3)	0.008
**Years of education**	15.0 (2.9)	16.5 (2.5)	< 0.001
**APOE ε4 (% at least one allele)**	67.27	28.62	< 0.001

**Fig. 1:**
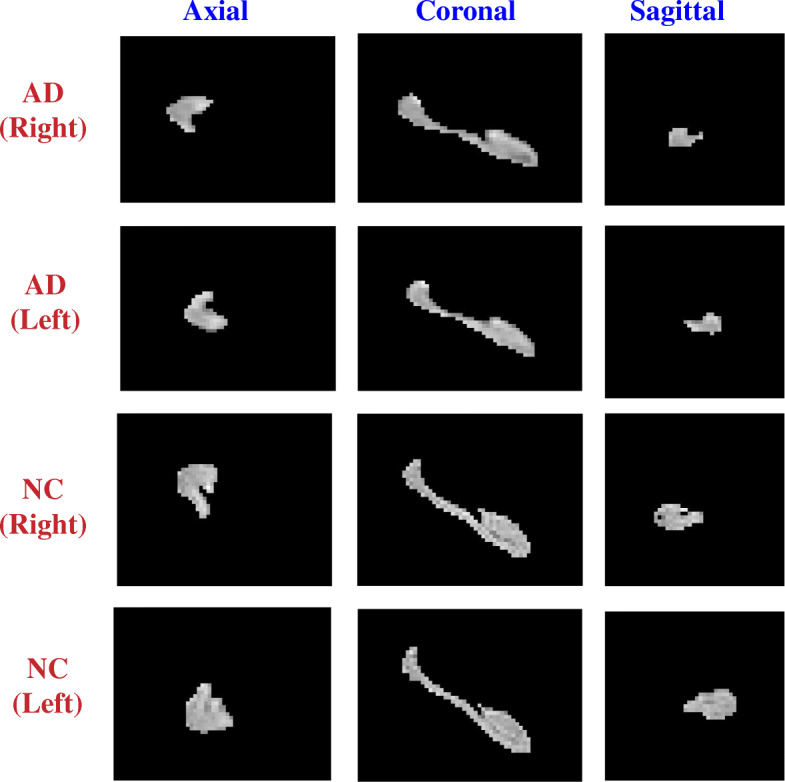
Examples of hippocampal segmentations (both left and right) from the AD and NC

There are two hippocampi (left and right) in the brain. We segmented left and right hippocampi using the very recent segmentation tool Hippmapp3r, which is based on 3D CNNs and robust for MRI images with brain atrophy and lesions associated with aging and neurodegeneration [37]. Hippmapp3r has shown to be producing accurate and fast hippocampal segmentations when compared to the existing segmentation algorithms [37]. Figure 1 shows examples of hippocampal segmentation results from the two groups AD and CN.

### Global shape representations

Global shape features, for each left (L) and right (R) hippocampus, were obtained using LB spectrum [25]. Compared to other shape descriptors such as spherical harmonics [24], LB spectrum is isometry-invariant and avoids pre-processing steps like mapping, registration, and alignment. Also, LB spectrum works for any Riemannian manifold [28], whereas spherical harmonics requires spherical parameterization, a smooth mapping from the surface to a unit sphere [27]. The shape and geometrical information of volumetric data can be discovered and extracted by taking the eigenvalues (i.e., the spectrum) of its LB operator. The LB spectrum can be considered as the set of squared frequencies that are associated with the eigen modes of a generalized oscillating membrane defined on the manifold [25]. Also, eigen values are invariant to isometric transformations and rely on the deformation applied to the boundary of the object [27]. Since, LB spectrum is isometric invariant; it is one of the most powerful ways to represent shape [25]. A brief detail of the LB spectrum is provided below.

Spectrum of the LB operator is defined for real-valued functions on Riemannian manifolds. For a real-valued function *f* defined on a Riemannian manifold *M*, the LB operator Δ is given as:

1 $$ \varDelta f=\operatorname{div}\ \left(\operatorname{grad}\ f\right) $$

with grad “*f*” being the gradient of “*f*” and the div being the divergence on the manifold*.* The LB operator is a linear differential operator and it can be calculated in local coordinates. LB operator is self-adjoint and semi-positive definite. It follows that the operator Δ admits an orthonormal eigensystem. It consists of eigenvalues λ_*i*_ ϵ ℝ and eigen function *f*_*i*_ pairs. More details of LB operator can be found in [28, 38]. The eigen values are called LB the spectrum and contain intrinsic geometrical information of the segmented data. Generally, normalized eigen values are utilized to obtain a scale-invariant global shape representation. This spectrum was calculated through finite element computations [38].

### Deep visual features of the hippocampus segments

**Fig. 2:**
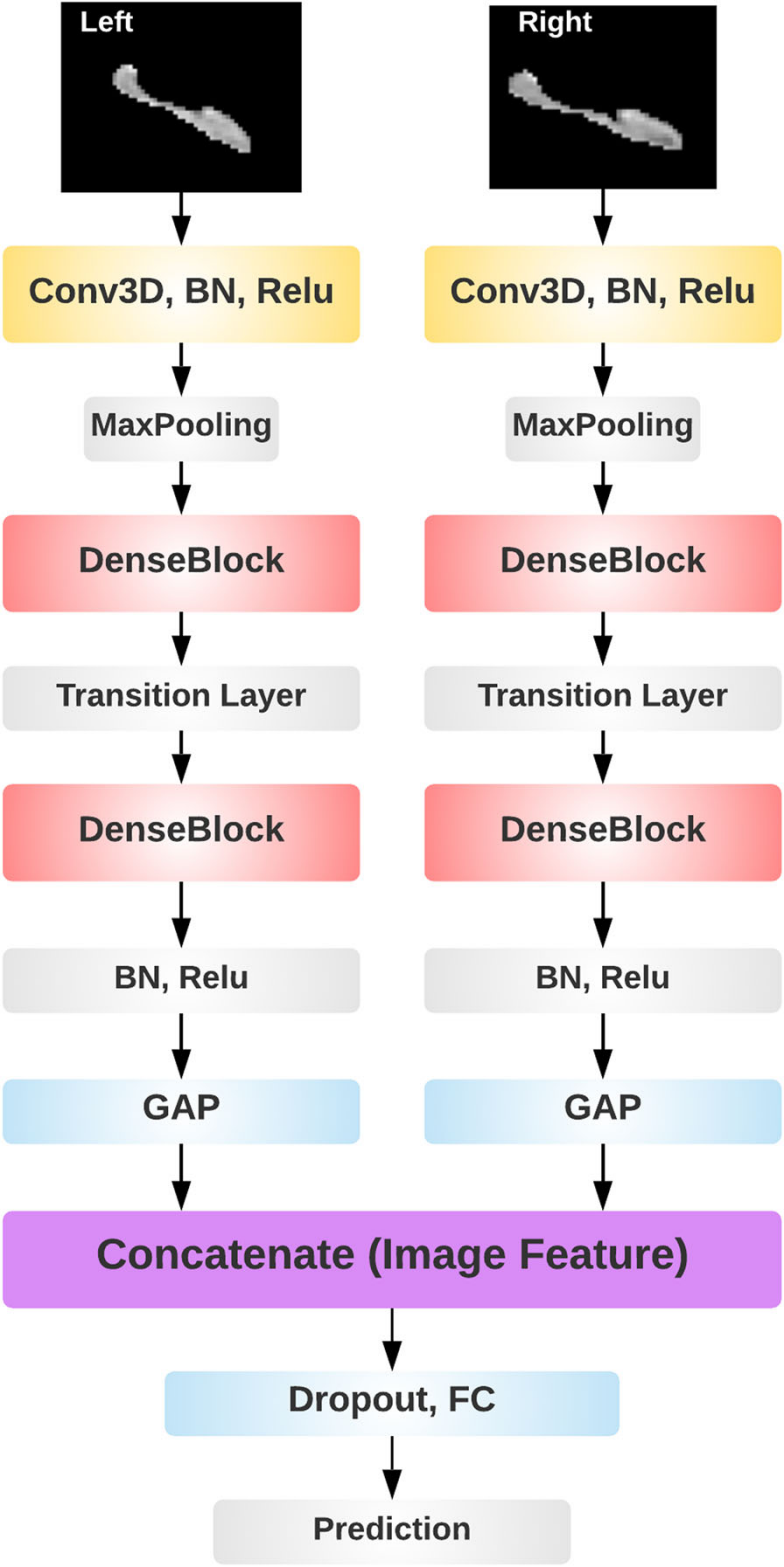
The architecture of DenseCNN

We obtained deep visual features of the hippocampus segments from DenseCNN, a deep convolution neural network model for AD classification that we have recently developed based on hippocampus segments [36]. DenseCNN has 3 dense layers, with each layer consisting of 2 convolutional layers, combined with Batch normalization (BN) layers and Relu activation layers. Transition layers end with a max pooling layer to decrease the size of input data. DenseCNN has two streams for left and right hippocampus segments correspondingly (Fig. 2). Each stream has an initial 3D convolutional layer followed by a BN layer and a Relu activation layer, extracting low-level image features. Then a max pooling was used to ignore 0 voxels on the edges of the input data and reduce the data size. Two dense blocks and a transition layer were stocked in each stream, using 8 and 16 filters correspondingly. At the end of each stream is a global average pooling (GAP) layer, which compresses high dimensional image features to 1-dimensional features. After the GAP layer, two streams were merged followed by a dropout layer. Finally, a fully connected layer and a SoftMax layer were used for generating prediction. The architecture of this model is shown in Fig. 2. The output of the last GAP layer is the CNN features considered here. For each left and right hippocampus, deep visual features were obtained after the last GAP layer of the DenseCNN.

### Joint training DenseCNN2 using DenseCNN features and global shape features

**Fig. 3:**
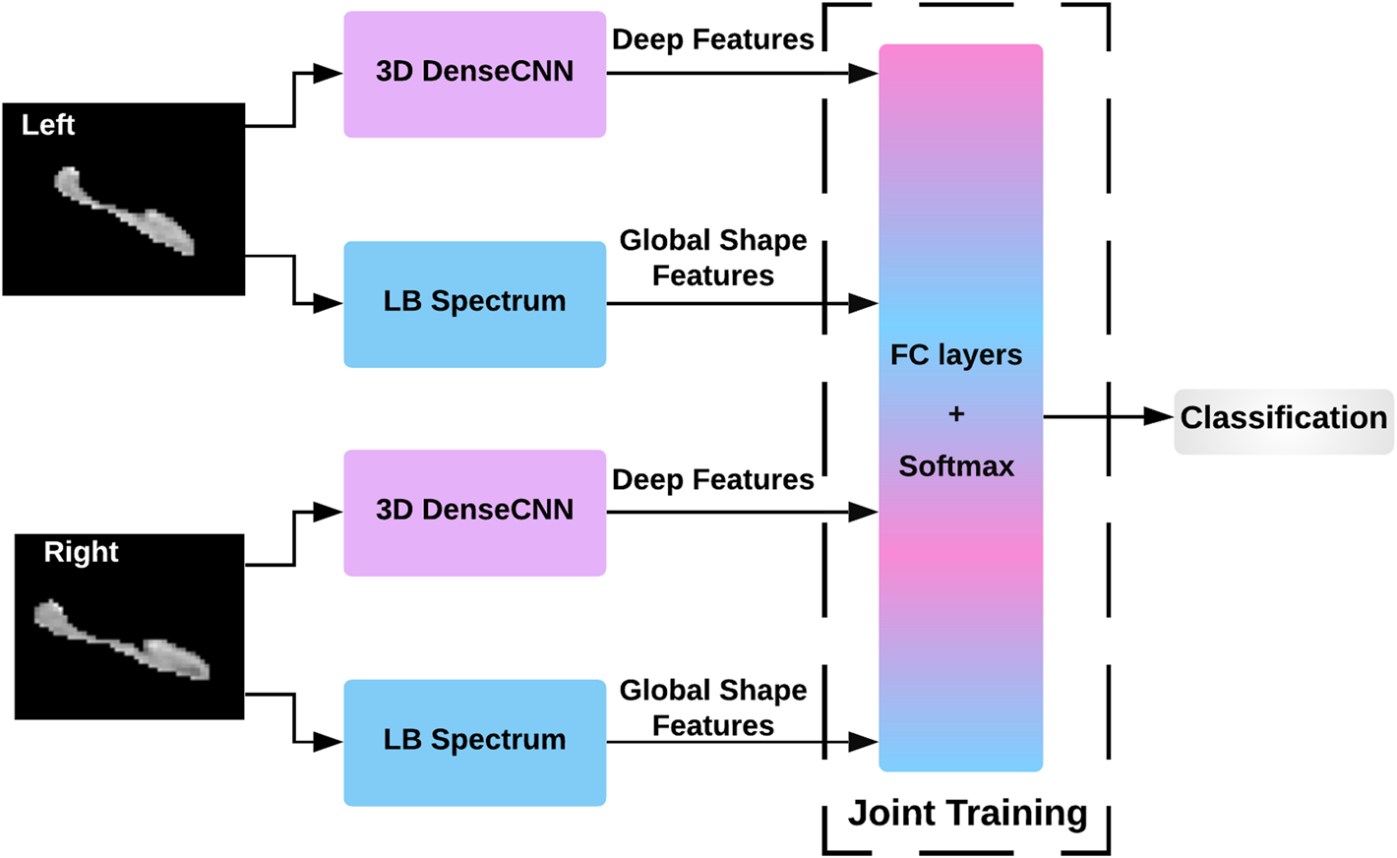
The structure of the joint model

Using the abovementioned methods, both shape and deep features were extracted using hippocampus segments. Global shape description for each of the left and right hippocampus is calculated using the LB spectrum (see the “Global shape representations” section) where “*f*” is the output of the segmentation tool *Hippmapp3r*. These two types of (shape and DenseCNN features) were expanded and connected together. The two parts of features re-trained in a neural network using a joint training strategy. The learned features from DenseCNN and shape features were combined by a network architecture with fully connected layers followed by softmax layer for AD classification. This joint training strategy can make better global optimization for two models. The performance of this joint model was analyzed by varying the number of fully connected layers and also varying the number of neurons in each layer. Both training and testing data were normalized using a *z*-score to combine deep features and shape features on the same scale. Figure 3 illustrates the architecture of direct combination of CNN features and shape features.

### Evaluation

To train and test the classification performance of the proposed DenseCNN2 model, a total of 326 AD subjects and 607 CN subjects were used. DenseCNN2 was implemented with python and Keras library on the Tensorflow backend. Fivefold cross-validation with validation and test set was used for model evaluation. Each time, 1-fold of the data set was used for testing, and 4 folds were further split into training and validation with the validation part consisting of 10% of the training data. This process was repeated 10 times and the average result was reported. In this experiment, as the spectrum of the LB operator contains intrinsic shape information, we considered the top 30 LB eigen values. Normalized eigen values were used and the type of normalization used here was λ_n_ → λ_n_/λ_1_ with logarithmic scale.

To evaluate the performance of DenseCNN2, five measures were computed for evaluation: overall accuracy, sensitivity, specificity, receiver operating characteristic (ROC) curve, and the area under ROC curve (AUC).

### Comparison with other models

The performance of DenseCNN2 with combined visual and shape features was compared with deep learning models with shape features alone and visual features alone (DenseCNN). We also compared DenseCNN2 with existing traditional and deep learning methods such as hippocampus volumes, CHF features [17], ResNet [39], 3D CNN [32], hybrid CNN-RNN [33], multi-model CNN [40], and 3D DenseNet [41]. For existing deep learning methods, the reported results are considered here. Overall accuracy, sensitivity, specificity, and AUC were compared.

### Data visualization using UMAP

We performed data visualization to demonstrate how combined visual and shape features contributed in separating AD from normal. Dimension reduction plots were used to visualize the data by placing similar data points in close proximity in a low-dimensional space. UMAP (Uniform Manifold Approximation and Projection), an effective tool for visualizing clusters or groups of data points and their relative proximities with nonlinear mapping [42], were used for data visualization. The quality of the separation was computed using three commonly used probabilistic class separability measures, namely, Jeffries-Matusita distance, Bhattacharya distance, and the transformed divergence [43]. The greater the values of these measures indicate better separation between the classes.

## Results

### Comparison of DenseCNN2 with combined visual and shape features with deep models with shape or visual features alone

DenseCNN2 was compared with deep learning model using shape features alone (DL_shape), and with DenseCNN with visual features. DL_shape was constructed using fully connected layers network with softmax layer classification. For training DenseCNN2 model, the network parameters were randomly initialized at the beginning. For learning deep features, batch-normalization has been performed in order to stop learning irrelevant features at convolutional layers and for faster training. Also, to avoid overfitting, the dropout and L2 regularization were used in the network. Optimal performance of the model is found with dropout factor of 0.5 and L2 weight decay of 0.02. The performance of the joint training with both (deep and shape) features was analyzed by varying the number of fully connected layers and also varying the number of neurons. The stochastic gradient descent optimizer was used with the initial learning rate of 1e−3. The momentum was set to 0.9 and the cross-entropy loss function was used. DenseCNN2 has achieved an accuracy of 92.52%, sensitivity of 88.20%, specificity of 94.95%, and AUC of 97.89%, which are better than DL_shape and DenseCNN (Table 2). DCNNs are able to encode the local shape features including local edge segments and relations. But, it is sensitive to how these local features fit together as a whole to represent global shape features. Thus, our combined model DenseCNN2 has better performance over DenseCNN.

**Table 2:** Performance comparison of DL_shape, DenseCNN, and DenseCNN2

Method	Accuracy	Sensitivity	Specificity	AUC
**DL_shape**	70.89	54.31	75.42	76.15
**DenseCNN**	89.91	84.91	94.01	96.42
**DenseCNN2**	92.52	88.20	94.95	97.89

**Fig. 4:**
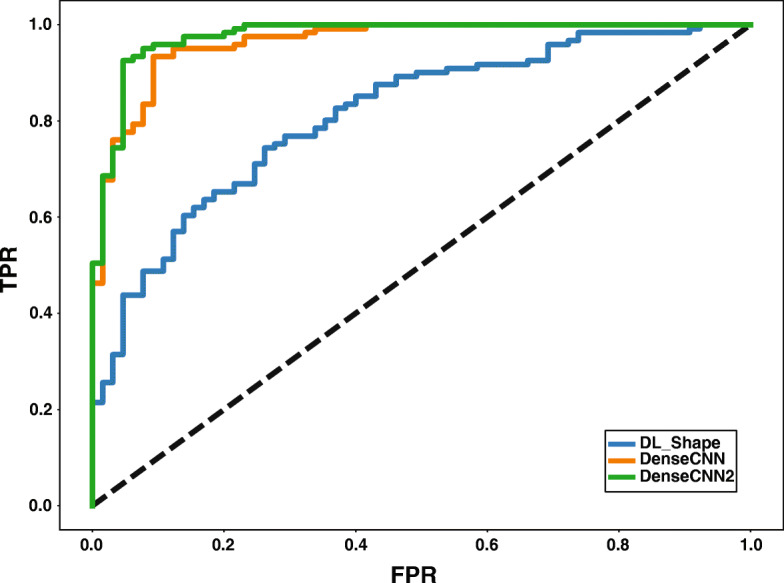
ROC curves of shape, DenseCNN, and DenseCNN2

Figure 4 illustrates the ROC curves of the shape and DenseCNN and DenseCNN2 for classifying AD vs. NC. These results demonstrate that combining CNN features with shape features in DenseCNN2 improved the performance of AD classification compared with CNN features or shape features alone**.** These results indicate that the model has learned complementary features for AD classification based on global shape and visual features of hippocampus segments. Thus, DenseCNN2 is better than DenseCNN by modeling the global shape features along with visual features.

### Comparison with other methods

The performance of the proposed model was compared with seven traditional and deep learning methods. Table 3 shows the comparison performances between DenseCNN2 and the existing methods. DenseCNN2 achieved a classification accuracy of 92.52%, a specificity of 94.85%, and an AUC of 97.89 for AD vs. NC classification, which is higher than both traditional and deep learning methods. Although we reported higher performance, the number of subjects and partitions of training, testing data are different with the existing methods. Compared to other models, DenseCNN2 is a lightweight 3D deep convolutional network model based on DenseCNN, with no particular feature engineering needed. DenseCNN2 does not heavily rely on data augmentation and is fast in training and prediction and has potential to be used for practical and clinical purposes. It integrates additional global shape information into the model for improved performance.

**Table 3:** Performance comparison of DenseCNN2 with other methods

Method	Accuracy	Sensitivity	Specificity	AUC
Hippo volumes	50.54	42.12	58.40	53.54
Hippo CHF features	85.12	76.31	81.40	–
3D CNN	86.94	79.36	93.21	86.40
ResNet	90.00	–	–	95.60
Hybrid CNN-RNN	89.17	84.64	93.16	91.00
Multi-model CNN	88.90	86.62	90.81	92.50
3D DenseNet	92.29	**90.63**	93.72	96.95
DenseCNN2	**92.52**	88.20	**94.95**	**97.89**

### Data visualization using UMAP with DenseCNN features and with DenseCNN2 features

**Fig. 5:**
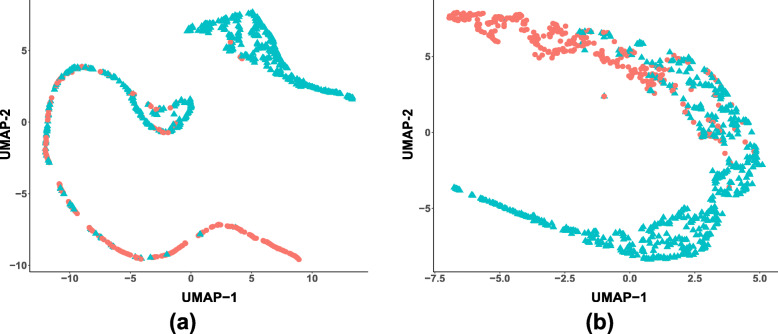
Two dimensional UMAP embedding with visual features (left) and with combined visual and global shape features (right). The data points in color red represent AD subjects and in color blue represent NC subjects

We used data visualization to get insights into the data and the discrimination information between classes. We demonstrated through 2D embedding of UMAP that the class discrimination was improved when shape features were added. Figure 5a, b provides the 2D embedding UMAP of hippocampus data with visual features and with combined visual and shape features respectively. Visually, the separation between the two classes with combined features is greater than the separation with visual features alone. In Fig. 5a, UMAP provided two well separated clusters, but many data points from different classes are overlapping and mis-classified. On the other hand, while there is overlap with combined features, but greater amount of the data is well separated (Fig. 5b).

Table 4 provides quantitative measures of separations for the UMAP with visual features and with combined features. It is clear that the values of separations increased for combined features compared with deep visual features alone. This indicates that the classes with combined features have more separation than those with deep visual features alone. These results further validated that the integration of shape features in deep learning model is necessary for AD classification based on MRI data.

**Table 4:** Comparison of class separability indices

Method	Jeffries-Matusita	Bhattacharryya	Divergence
**Visual features**	1.74	2.04	281.30
**Combined visual and shape features**	1.88	2.85	324.33

## Discussions and conclusion

We proposed a lightweight 3D deep convolutional network model, DenseCNN2, for AD classification using combined hippocampus segmentations and global shape representations. We demonstrated that the combination of deep features and global shape features improved the performance of classifying AD from normal. Also, it is observed through 2D embedding of UMAP that the class discrimination is improved when shape features are added. Also, DenseCNN2 is compared with existing traditional and deep learning-based methods. It is performing better or comparable with all the existing methods.

Future works are warranted to further improve and extend the current study. First, the performances were heavily relied on high-quality hippocampus segments. However, current hippocampus segmentation tools are not ideal. We will develop more robust segmentation algorithms for the hippocampus. We can also avoid this problem by building deep learning models on other relevant regions or whole brain MRI, since brain segments on average have much higher quality than hippocampal segments. Second, while DenseCNN2 is a lightweight model without the need of particular feature engineering and data augmentation and is fast in training and prediction, the data preprocessing step was extensive including segmentation, normalization, and multiple extractions, which needed a well understanding of the dataset and expertise in image processing. In our future study, we will investigate the possibility of building an end-to-end deep learning model, to simplify the whole process of data preprocessing, model training and testing, and data visualization. Third, our model is based on only MRI data. It is known that AD risk is also affected by genetic variants, demographics, comorbidities, medications, socio-economic determinants among others. So, our future study would be to develop multi-modal prediction models for AD classification by combining the MRI data with other data available in ADNI. We will also investigate how patient genetics, demographics, medications, and comorbidities are involved in AD etiology by examining visual and shape changes in the hippocampus. Studying such correlations or interactions provides important insights for AD initiation and progression. Fourth, though our dataset consisted of 326 AD subjects and 607 control normal (CN) subjects from the ADNI database, it still may not be large enough to guarantee generalizability. Further study could utilize more samples from ADNI, or other datasets such as Oasis (http://www.oasis-brains.org). And we believe more new samples should be collected, since new MRI samples can test the generality of existing AD detection approaches.

## Limitation

Limitations include the following: (1) current model was trained solely on hippocampus regions. Other brain regions could be used for further improvements; (2) current model used only MRI data. Other data types including clinical, genetic, and genomics will be incorporated in our future studies; (3) only ADNI data was used for both model training and testing. Other independent datasets could be used for both model improvement and independent testing; (4) this study focused on classifying AD versus normal control. We will adopt the “light” model approach demonstrated in this study to classify AD versus MCI versus normal, which is a more challenging task than classifying AD versus normal.

### Data availability

Features and code for DenseCNN2 are publicly available at http://nlp.case.edu/public/data/DenseCNN2.

## Data Availability

The MRI data used in this study was from Alzheimer’s Disease Neuroimaging Initiative (ADNI).

## Abbreviations

MRI: Magnetic resonance imaging
DCNN: Deep Convolution Neural Networks
LB: Laplace Beltrami
AD: Alzheimer’s disease
NC: Normal control
UMAP: Uniform Manifold Approximation and Projection
